# Genome-wide identification and characterization, phylogenetic comparison and expression profiles of *SPL* transcription factor family in *B*. *juncea* (Cruciferae)

**DOI:** 10.1371/journal.pone.0224704

**Published:** 2019-11-05

**Authors:** Jian Gao, Hua Peng, Fabo Chen, Yi Liu, Baowei Chen, Wenbo Li

**Affiliations:** 1 Department of Life Sciences and Technology, Yangtze Normal University, Fuling, Chongqing, China; 2 Centre for Green Development and Collaborative Innovation in Wuling Mountain Region, Yangtze Normal University, Fuling, Chongqing, China; 3 Sichuan Tourism College, Chengdu, Sichuan, China; Huazhong University of Science and Technology, CHINA

## Abstract

SQUAMOSA promoter-binding protein-like (SPL), as plant specific transcription factors, is involved in many plant growth and development processes. However, there is less systematical study for *SPL* transcription factor in *B*. *juncea* (Cruciferae). Here, a total of 59 *SPL* genes classified into eight phylogenetic groups were identified in *B*. *juncea*, highly conserved within each ortholog were also found based on gene structure, conserved motif, as well as clustering level. In addition, clustering of SPL domain showed that two zinc finger-like structures and NLS segments were identified in almost of *BjuSPLs*. Analyzed of putative cis-elements for *BjuSPLs* demonstrated that *SPL* transcription factors were involved in adverse environmental changes, such as light, plant stresses and phytohormones response. Expression analysis showed that differentially expressed *SPL* genes were identified in flower and stem development of Cruciferae; such as *BjuSPL3a-B*, *BjuSPL2b_B* and *BjuSPL2c_A* were significantly expressed in flower; *BjuSPL 3b_B* and *BjuSPL10a_A* were significantly expressed in stem node (VP: vegetative period). Moreover, 28 of the 59 *BjuSPLs* were found involved in their posttranscriptional regulation targeted by *miR156*. We demonstrated that miR156 negatively regulated *BjuSPL10a_A* and *BjuSPL3b_B* to act for stem development in *B*. *juncea*.

## Introduction

Plant SQUAMOSA promoter-binding protein-like (SPL), acts as fundamental roles in plant growth and development, and are defined by the SBP domains (for SQUAMOSA-PROMOTER BINDING PROTEIN) that have a highly conserved regions of 76 amino acids in length. SPL contains a conserved SBP (SQUAMOSA promoter binding protein) domain having two zinc-binding sites consisted of Cys-Cys-Cys-His and Cys-Cys-His-Cys, respectively, and a nuclear localization signal (NLS) motif located at the C-terminal of the SBP domain [1]. Researches have reported that the SBP domain can act as both nuclear import and sequence-specific DNA binding to a consensus-binding site through GTAC core motif and gene-specific flanking regions [2,3]. *SPL* genes are widely distributed in gymnosperms, mosses, single-celled green algae and angiosperms [4], *SPL* genes were firstly detected in *Antirrhinum majus* that were involved in the control of early flower development by binding to the promoter of the floral meristem identity gene *SQUAMOSA* (*SQUA*).

In recent years, numerous studies have characterized *SPL/SBP-box* gene family based on genome-wide identification in many plant species [5–19]. Sixteen *SBP-LIKE* (*SPL*) genes were clustered into eight clades following their conserved SBP domain in *Arabidopsis* [1]. Of those, 10 SPL genes belong to five clades are targeted by *miR156* [2, 3], which might highly conserved and acted as important regulators in vegetative phase change in plants [4, 5].

Moreover, *SPL* genes played vital regulatory roles in diversified plant developmental processes in different plant species, such as vegetative phase change [6], male fertility [7], GA biosynthesis [8], plant architecture [9] and response to stress [9] in plant. However, genome-wide analysis of *SPL* genes is scarcely in *B*. *juncea* owing to lack of genome information, compared with other plant species.

*B*. *juncea* (Cruciferae), the tumorous stem mustard, was famous as the raw material for Fuling mustard in China. Currently, the genetics breeding, physiology, biochemistry and classification of *B*. *juncea* (Cruciferae) have been extensively studied, but little work has been done at the molecular level. Transcription factors *SPL* and their regulation roles in *B*. *juncea* are poorly understood. Here, genome-wide analysis and molecular dissection of the *BjuSPL* gene family were performed in this study, together with their chromosomal locations. In addition, we constructed phylogenetic tree for *SPL* gene family collected from *A*. *thaliana*, *B*. *juncea* and *B*. *napa*. Moreover, cis-acting elements, conserved motif, gene structure and expression pattern of all identified *BjuSPL* genes in *B*. *juncea* were also systematically analyzed. This study will give insight into the structure and evolution of the *SPL* genes family in *B*. *juncea*, thereby further helpful for molecular function analysis of those *SPL* genes in the future.

## Methods

### Sequence sources

*Brassica juncea* sequences were kindly provided from Jinhua Yang of Zhejiang University. We retrieved the *SPL* protein sequence of *A*. *thaliana* and *B*. *napa* from the Plant Transcription Factor Databases 55 (Plant TFDB v4.0, planttfdb.cbi.pku.edu.cn/) [10], together with their General Feature Format (GFF) file were obtained from Arabidopsis Information Resource (TAIR release 10, http://www.arabidopsis.org), and (http://www.genoscope.cns.fr/brassicanapus/data/Brassica_napus.annotation_v5.gff3.gz), respectively. Name and gene IDs of 17 and 59 known *SPL* genes in *A*. *thaliana* and *B*. *napa* were shown in S1 Table, respectively.

### Identification and distribution of *SPL* transcription factor family in *B*. *juncea*

SBP domain (PF03110) for SPL transcription factor was downloaded from Pfam [11] (Pfam; http://pfam.xfam.org/), thereby exploiting for the identification of all possible *SPL* genes from *B*. *juncea* using HMMER (v3.1b2)[12] (http://hmmer.org) through the e-value <1e-10. All non-redundant hits with expected values were collected and then compared with the *SPL* family in PlnTFDB (http://plntfdb.bio.uni-potsdam.de/v3.0/) and PlantTFDB (http://planttfdb.cbi.pku.edu.cn). After that, each putative *SPL* gene was confirmed to the presence of SBP domain using SMART [13] (http://smart.embl-heidelberg.de/), CDD [14] (http://www.ncbi.nlm.nih.gov/Structure/cdd/wrpsb.cgi) and Inter-ProSca (http://www. ebi.ac.uk/Tools/InterProScan/). The theoretical MW (molecular weight) and PI (isoelectric point) of *BjuSPLs* were investigated through Expasy57 (http://web.expasy.org/protparam/). We fetched the physical location information of the each *SPL* from the corresponding GFF files. Subsequently, we used mg2c (http://mg2c.iask.in/mg2c_v2.0/) to depict their distribution in each *B*. *juncea* chromosome. In addition, we used BLAST-searching [15] for *BjuSPLs* against each other to identify the duplicated *BjuSPLs* genes, which were defined when both their identity and query coverage was > 80% of their partner sequence [16]. Tandem-duplicated genes were identified as an array of two or more homologous genes within a distance of 100 kb. A chromosome region containing more than two genes within 200 kb was defined as a gene cluster [17]. Moreover, comparison and annotation of orthologous gene clusters among *B*. *juncea* and *B*. *napa* were conducted using orthoVenn software [18].

### Phylogenetic analysis of *BjuSPLs*

For phylogenetic analysis of multiple sequence, full-length protein sequences of *BjuSPLs* were performed by ClustalW alignment, and then a phylogenetic tree was constructed with MEGA 7.0 software [14] (https://mega.nz/) by the Neighbor-Joining (NJ) method, carried out with 1000 replicates boot-strap test.

### Gene structure and conserved motif of *BjuSPLs*

Gene structure display server (GSDS 2.0) [19] program (http://gsds.cbi.pku.edu.cn/) was used to illustrate the exon/intron structures of *BjuSPLs* through inputting their GFF files. MEME (suite 4.11.4) [20] (http://meme-suite.org/) was used for elucidation of conserved motifs of *BjuSPLs*. MEME was run locally through the parameters as follows: optimum width of motif: 6–250 and maximum number of motifs: 9. In addition, MAFFT version 7 (http://mafft.cbrc.jp/alignment/server/) was selected for presenting SBP domain. And we used the weblogo platform to generate sequence logos (http://weblogo.berkeley.edu/).

### *Cis*-acting elements analysis of promoter regions from *BjuSPLs*

To investigate the putative cis-acting elements of 59 candidate *BjuSPLs* genes, their promoter sequences (2,500 bp upstream of the initiation codon “ATG”) were extracted from genome sequences of *B*. *juncea* and then conducted using PlantCARE [21] (http://bioinformatics.psb.ugent.be/webtools/plantcare/html/search_CARE.html).

### Sample collection and RNA isolation

The *B*. *juncea* ‘YA1’, ‘YA2’ and ‘YA3’ were cultivated in a greenhouse at the experimental farm of the Yihe (Yangtze normal university experiment base) in 2018. Firstly, we sowed seeds of *B*. *juncea* ‘YA1’, ‘YA2’ and ‘YA3’ in sterilized soil for 2 weeks under normal growth conditions (23°C, 16 h light/8 h dark). After that, 2-week-old plants were transferred and kept for 15 days in the cold room (5 ± 1°C, 12 h light/12 h dark) for vernalization treatment. After the vernalization periods, the plants were grown in a normal growth room under normal growth conditions (23°C, 16 h light/8 h dark). At least three independent biological replicates for leaf of seedling stage (SS), leaf and stem of flowing period (FP), leaf and stem of mature period (MP) from *B*. *juncea* ‘YA1’, ‘YA2’ and ‘YA3’ were collected for RNA-Seq, as well as qRT-PCR for the candidate differential expression *BjuSPLs* with three replicates. Of those, samples comprised of seedling, leaf (vegetable periods), stem nodule (vegetable periods), stem nodule (Flowing periods), flower and legume from ‘YA1’ were collected for qRT-PCR for miRNAs and their related targets with three replicates. All harvested tissue were immediately frozen in liquid nitrogen and stored at -80°C for qRT-PCR respectively. Subsequently, using the mirVana^™^miRNA Isolation Kit (Ambion) and Trizol Reagent (Invitrogen, Nottingham, UK) kit following the manufacturer’s instructions, small RNA and total RNA was isolated from each sample.

### Expression pattern analysis of candidate *BjuSPLs* in *B*. *juncea*

For analyze the expression patterns of *BjuSPLs* genes, our private available RNA expression profile data including leaf of Seedling stage (SS), leaf and stem of flowing period (FP), leaf and stem of mature period (MP) from YA1 (yonganxiaoye1), YA2 (yonganxiaoye2) and YA3 (yonganxiaoye3) were selected respectively (S2 Table). We used Log2 (FPKM) for calculating the expression levels of *BjuSPL* genes and their expression patterns were clustered by hierarchical clustering model and illustrated using homemade R language.

### Prediction of *BjuSPLs* targeted by *miR156*

*BjuSPLs* targeted by *miR156* collected from literature were predicted using psRNATarget [22](http://plantgrn.noble.org/psRNATarget/?function.3), and the parameters were set as follows: with the maximum expectation of 3 and the target accessibility (UPE) of 50.

### Validation of candidate *BjuSPLs* in *B*.*juncea* using real-time qRT-PCR

To monitor the candidate *BjuSPLs* in *B*. *juncea*, ten candidate *BjuSPLs* (*BjuSPL10b_A*, *BjuSPL10a_A*, *BjuSPL10b_B*, *BjuSPL11a_B*, *BjuSPL11b_B*, *BjuSPL13a_B*, *BjuSPL2a_A*, *BjuSPL2b_B*, *BjuSPL2c_B*, *BjuSPL3c_A*, *BjuSPL7a_B and BjuSPL9b_B* H) were selected for qRT-PCR (ABI 7500 real-time PCR System, United States) based on their expression patterns **(S2 Table).** The primers are designed by Primer 5.0 software for qRT-PCR experiments and *B*. *juncea* gene (Actin) is used as a standard control (**S3 Table**). First-strand cDNA synthesis was performed with 1 μg total RNA from ‘Yonganxiaoye 1’ *B*. *juncea* using a M-MLV reverse transcriptase (Promega). The amplification programs were performed according to the standard protocol of the ABI7500 system, and conducted in triplicate as mentioned by Jian et al. [23]. The relative quantitative method (2^-ΔΔCT^) was used to calculate the fold change in the expression levels of target genes [24].

### Validate the Candidate miRNAs by qRT-PCR

To validate of putative miRNAs targeted the candidate *BjuSPLs*, miR156 was selected based on their corresponding *BjuSPLs* comprised of *BjuSPL10a_A*, *BjuSPL10b_B* and *BjuSPL3b_B*. The primers are designed by Primer 5.0 software for qRT-PCR experiments and radish gene (Actin) is used as a standard control (S2 Table). Firstly, using One Step miRNA 1st cDNA Synthesis Kit (Shenggong, Chengdu, China), microRNA reverse transcription reactions were performed and incubated in an Eppendorf Mastercycler (Eppendorf North America, Westbury, NY) for 60 min at 37°C, followed by 5 min at 95°C, and then 4°C until further use. The qRT-PCR reactions were performed in a 10 μL volume containing 1 μL diluted reverse transcription product, 1 uL PCR buffer, 0.2 mM dNTPs, 2.0 U EasyTaq DNA polymerase (TransGen Biotech, Beijing, China), and 0.5 μM specific primer of miRNA156 (5-TTGACAGAAGAGAGTGAGCAC-3) and universal primer (5-TTACCTAGCGTATCGTTGAC-3) on Eppendorf Mastercycler. The amplification programs were performed according to the standard protocol of the ABI7500 system, and conducted in triplicate as mentioned by Jian et al.[23]. The relative quantitative method (2^-ΔΔCT^) was used to calculate the fold change in the expression levels of target genes [24].

## Results

### Genome-wide identification and distribution analysis of the *SPL* gene family in *B*. *juncea*

**Fig 1:**
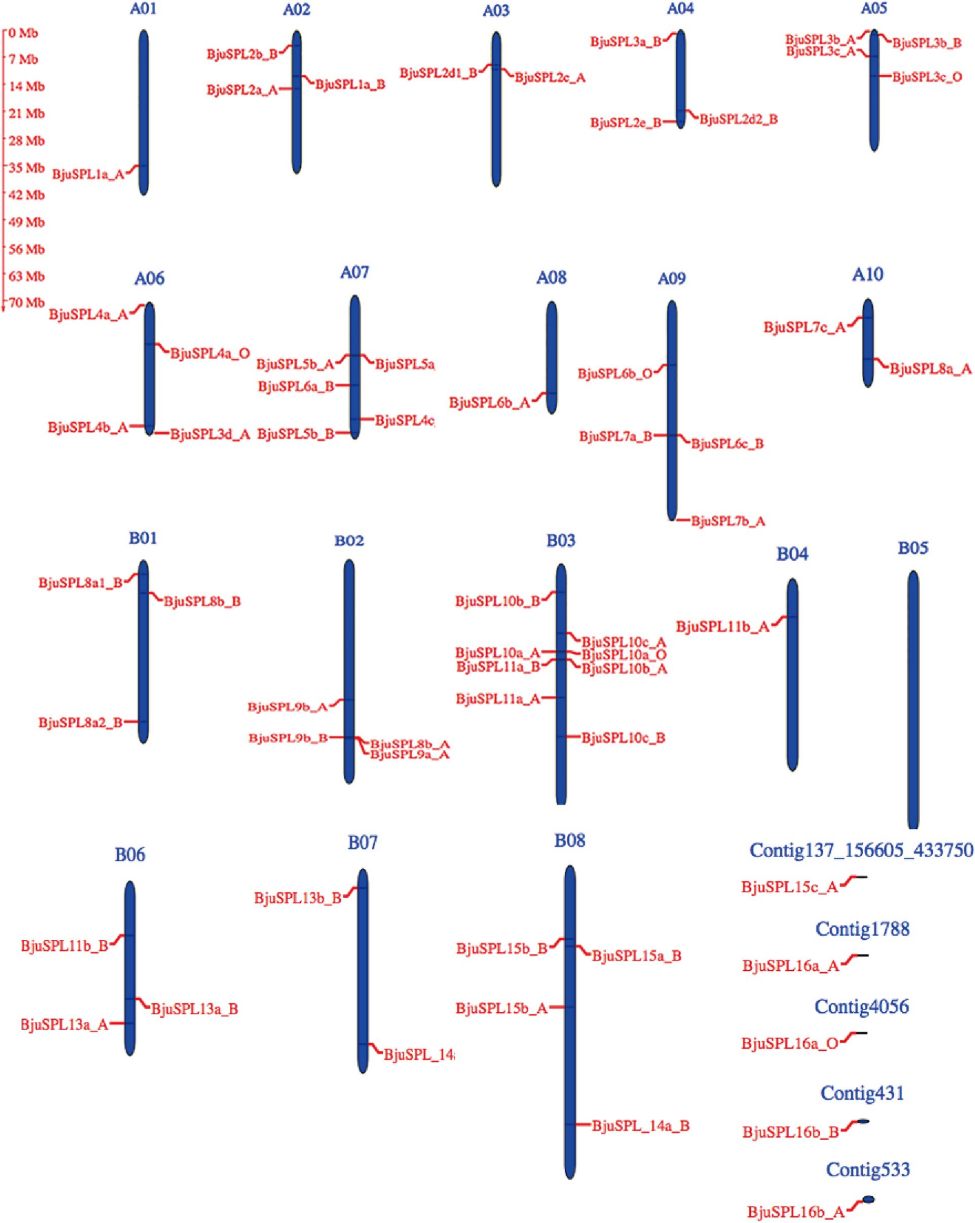
Distribution of *SPL* genes in the cruciferae genome. Chromosome distribution of *SPL* genes in *B*. *juncea* was identified, and the locations of closely linked genes were magnified. The chromosome number is indicated at the top of each chromosome. The scale is in megabases (Mb).

We used SBP domain (PF03110) to search against protein of *B*. *juncea* using HMMER software to identify *SPL* genes in *B*. *juncea* [12]. All non-redundant hits with expected values were collected and then compared with the *SPL* family in PlnTFDB (http://plntfdb.bio.uni-potsdam.de/v3.0/) and PlantTFDB (http://planttfdb.cbi.pku.edu.cn). In addition, we verified the presence of the SBP domain for the candidate *SPL* genes through SMART, Prosca and CDD[13, 14]. Total of 59 *SPL* genes were identified in *B*. *juncea* (**S4 Table**). Of those, we accurately named *BjuSPL* genes following their closest orthologs in *A*. *thaliana* and we coded their different paralogs as a, b, c, and so on, together with their order of the homologous chromosomes. The results showed that lengths and predicted molecular weight (Mw) of 59 *SPL* genes encoding protein ranged from 106 to 1,038 amino acids, as well as 12.161 to 114.796 kDa in *B*. *juncea* respectively (**S4 Table**). In addition, chromosome location showed that those *BjuSPL* genes were distributed in 17 of 18 chromosomes present in the *B*. *juncea* genome (A1-A10, B01-B04, B06-B08), as well as 5 contigs comprised of Contig533, Contig431, Contig4056, Contig1788, Contig137_156605_433750). We found that the numbers of *BjuSPL* genes mapped on each chromosome were uneven and ranged from 0 to 8. Of those, 8 *BjuSPL* genes were distributed in B08 chromosomes in *B*. *juncea*, but we did not detect *SPL* gene in B05 of chromosome (**Fig 1, S4 Table**). In other species, segmental duplication, tandem duplication, and polyploidization were identified determined the genomic locations of the SPL gene family. We identified 17 pairs of segmentally duplicated and three pairs of tandem-duplicated SPL genes in the *B*. *juncea* genome (S1 Fig). In addition, relationship between *BjuSPLs* with its homologs in *B*. *napus* were analyzed using OrthoVenn2 software, the results showed that the species of SPLs were identified obtained from 26 clusters, of those, 12 orthologous clusters were found shared between *B*. *juncea and B*. *napus*, expect for 14 single-copy gene clusters **(S5 Table)**.

### Phylogenetic analysis of *SPL* gene family in *B*. *juncea*

**Fig 2:**
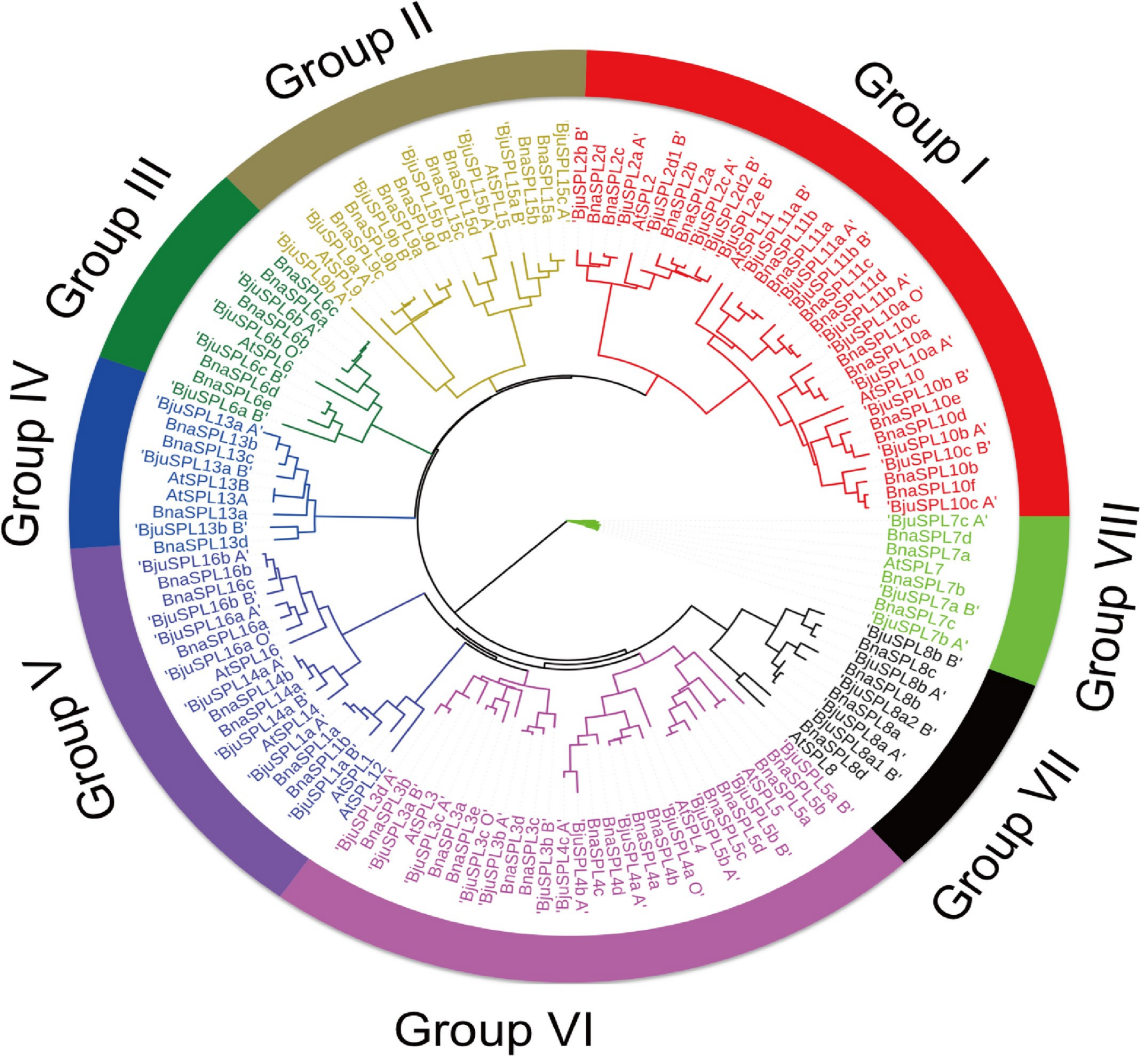
Unrooted phylogenetic tree of the *SPL* genes from different species (*B*. *juncea*, *A*. *thaliana* and *B*. *napa*). The SBP domain was identified and the phylogenetic tree was constructed using MEGA 7, and the maximum likelihood method with 1000 bootstraps. Different groups of the SBP family are divided with different colors.

Total of 135 *SPLs* (comprised of 59 obtained from this research in *B*. *juncea*, 17 from *A*. *thaliana* and 59 from *B*. *napa*) were used to construct a Neighbour-Joining (NJ) phylogenetic tree using MEGA 7.0 software. We found those *SPL* genes were clustered into 8 sub-groups named (I to VIII), and we found at least one protein was obtained from two species (*A*. *thaliana* and *B*. *napa*) for each group, with differently distinguished in *B*. *juncea* for their SPL orthologs. Mustard *SPL6*, *SPL7*, *SPL8* and *SPL13* were categorized into group III, VII, VIII and IV, respectively. Mustard *SPL5* and *SPL9* were categorized into group II, *SPL3*, *SPL4* and *SPL5* were categorized into group VI. Mustard *SPL2*, *SPL10* and *SPL11* were categorized into group I, and mustard *SPL1*, *SPL14* and *SPL1*6 belong to group V. However, *SPL12* were not identified in *B*. *juncea* in this study **(Fig 2)**.

### Gene structure and conserved motif analysis of *SPLs* in *B*. *juncea*

**Fig 3:**
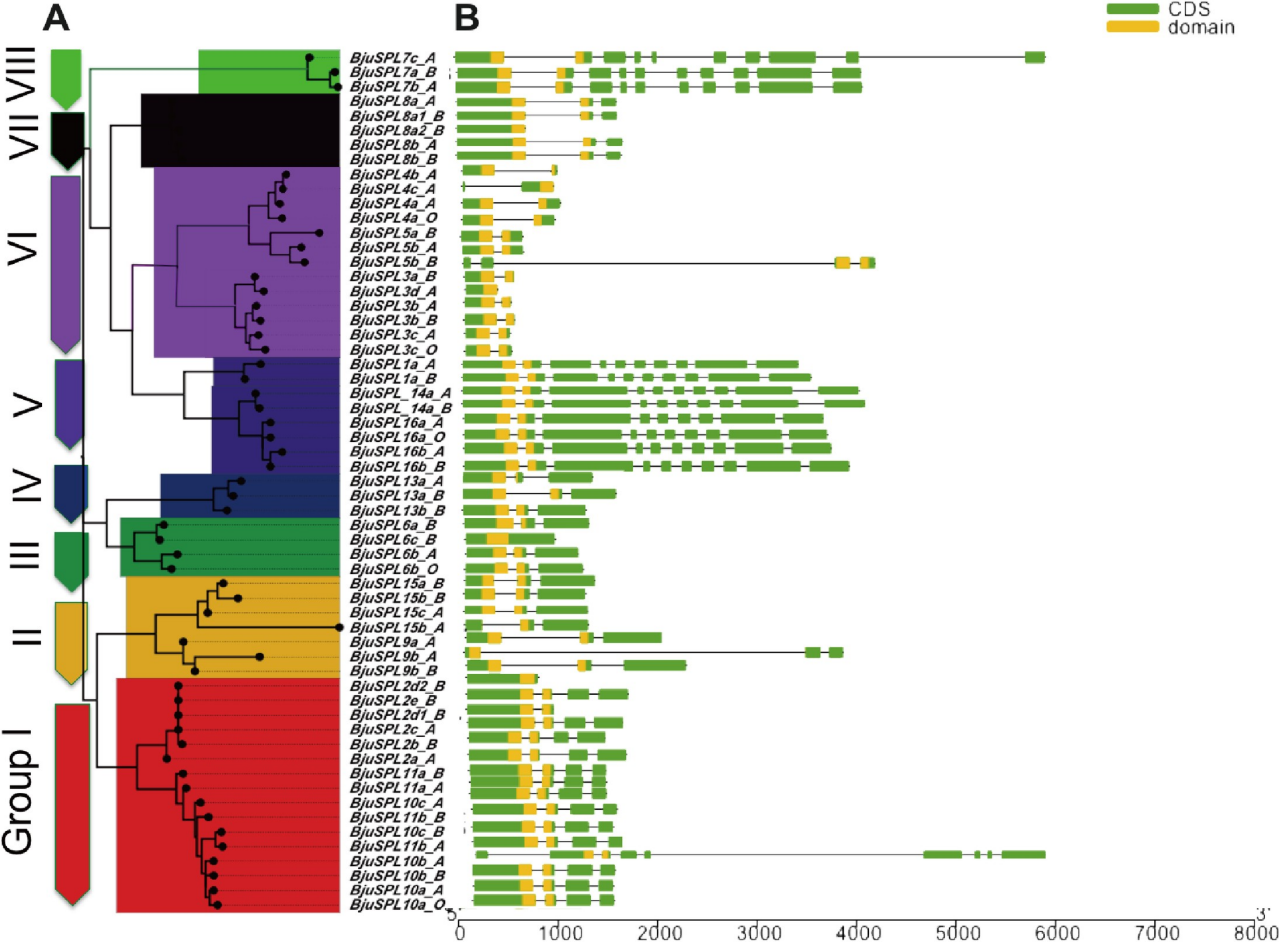
Phylogenetic tree of the *SPL* genes and genomic organization of *SPL* genes in *B*. *juncea*. A: The SBP domain was identified and the phylogenetic tree was constructed using MEGA7, and the maximum likelihood method with 1000 bootstraps. Different groups of the SBP family are divided with different colors. B: Exon/intron structures of *B*. *juncea SPL* genes. Untranslated 5′ and 3′ regions and CDS are shown in gray and black. Black lines connecting two CDS represent introns.

To demonstrate the structural diversity of the *B*. *juncea* SPL genes, conserved motif, exon/intron structure and putative *cis*-acting elements from *BjuSPLs* promoters were analyzed. We constructed unrooted NJ tree only using protein sequences of 59 *BjuSPLs* (**Fig 3A**), as well as their gene structures were analyzed by GSDS 2.0 and displayed in **Fig 3B**. The results showed that introns number varied from 2 to 12 for those 59 *BjuSPLs*. Of those, 20 *BjuSPLs* genes had 5 introns (5 *BjuSPL2*, 3 *BjuSPL6*, 4 *BjuSPL8*, 2 *BjuSPL9*, 3 *BjuSPL13* and 3 *BjuSPL15*); 6 *BjuSPL10* and 4 *BjuSPL11* had 6 introns, expect for *BjuSPL10b_A*, which contained 10 introns owing to segment duplication of some introns; More interestingly, *BjuSPL3*, *BjuSPL4* and *BjuSPL5* contained 2–3 introns, and we found *BjuSPL1*, *BjuSPL14* and *BjuSPL16* contained 12 introns with an ankyrin (ANK) domain along with an SBP domain, suggesting that these proteins represented different functions compared with other groups, which may play a role by interacting with other proteins in plant cells. We revealed that different *BjuSPLs* orthologs were similar to Arabidopsis orthologs and exhibited different exon-intron structures.

Further, protein motifs were identified for *BjuSPLs* through full-length proteins to clarify the characteristics of the SBP domain in *B*. *Juncea* (**S1 Fig**). The results showed that 20 motifs with the diversity of sequence structures recognized in *BjuSPLs* proteins among *BjuSPLs*, named motifs 1 to 20 were identified (**S2 Fig**). The lengths of 29 (motif 7) and 50 amino acids (motif1-6 et.al) were identified in those conserved motifs. The number of the conserved motifs varied from 3 for *BjuSPL2b_B* to 17 for *BjuSPL1a_B* respectively. Of those, we identified that 59 *BjuSPLs* proteins covered two Zn-finger like structure motif, and 56 *BjuSPLs* covered nuclear localization signal motif, except for *BjuSPL4b_A*, *BjuSPL4c_A* and *BjuSPL2_B*. Interestingly, most of *BjuSPL* proteins contained more motifs in SBP domain, expect for *BjuSPL15b_A* (had motif 1, 2 and 15), such as *BjuSPL2* (6–8 motifs), *BjuSPL7* (6–7 motifs), *BjuSPL14* (9–10 motifs) and *BjuSPL1* (11–13 motifs).

Moreover, we presented the SBP domain structures by constructing the multiple alignments of all 59 *BjuSPLs* proteins using MAFFT version 7. The results revealed that two zinc finger-like structures (Cys3His-type, Cys2HisCys), together with NLS segments were identified in all *BjuSPLs*, except for three SPLs (*BjuSPL4b_A*, *BjuSPL4c_A* and *BjuSPL2B2_B)* without NLS (**S3 Fig**). We demonstrated that two Zn-finger structures and NLS section in the sequences SBP domain of *BjuSPLs* were conserved in *B*. *juncea*, resulted from a similar number and type of SBP motifs identified in each of *BjuSPL* orthologs. We concluded that similar gene structure, motif architecture of *BjuSPL* orthologs were significantly clustered into the same phylogenetic tree.

### The *cis*-acting elements analysis of *BjuSPLs* gene promoter regions

To investigate putative functional of 59 *BjuSPL* genes in *B*. *juncea*, we collected their upstream sequences (2,500 bp upstream of the initiation codon) and then used for cis-acting element prediction by PlantCARE. More than 45 types of putative cis-elements were identified present in the promoters of *BjuSPLs*, such as light responsive elements, stress responsive elements, phytohormone response element, gibberellin, salicylic acid (TCA-element) and MeJA (**S4 Fig, S6 Table**). This suggests the most of *BjuSPLs* genes were involved in different biological processes, including response to abiotic stresses (defense and stress) and phytohormones in *B*. *juncea*.

### Expression profiles of *SPL* genes in *B*. *juncea*

**Fig 4:**
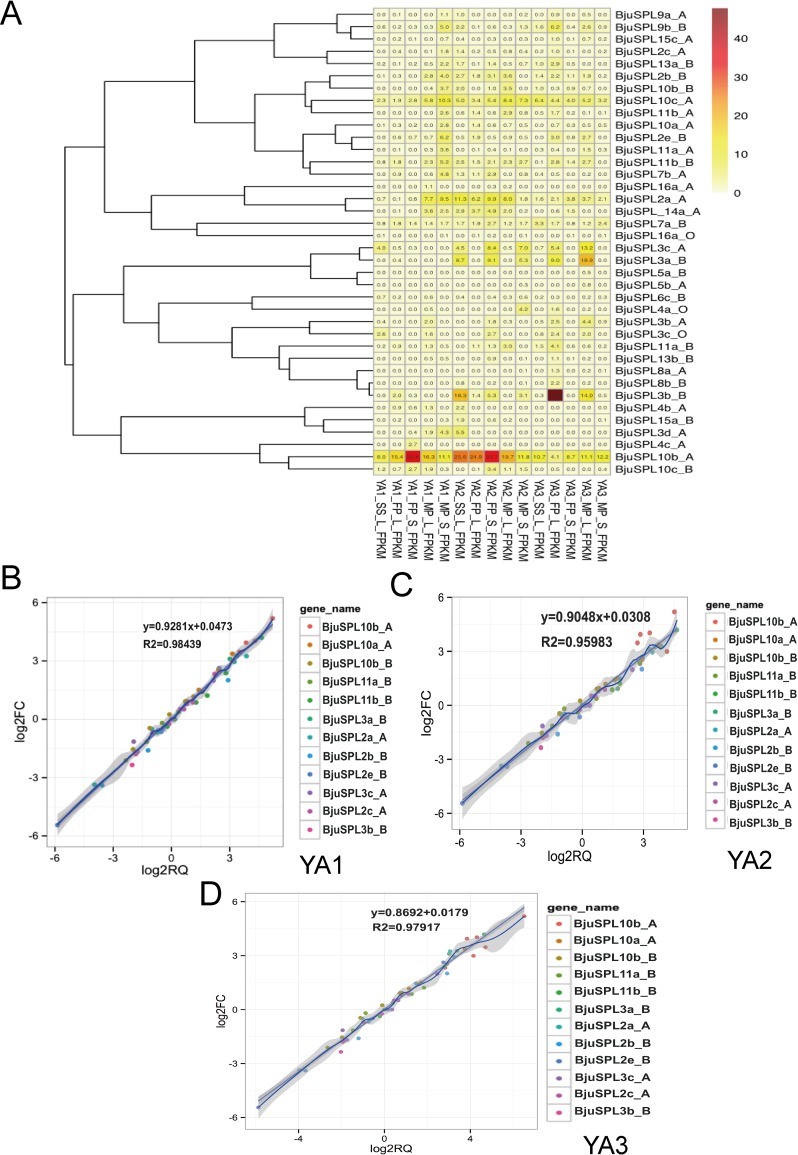
Expression patterns of *BjuSPL* genes and validate of candidate *BjuSPLs* in different tissues and cultivars of *B*. *juncea*. A. Expression patterns of *BjuSPL* genes in different tissues and cultivars of *B*. *juncea*. The color represents *BjuSPLs* expression levels: Log2 (FPKM). The phylogenetic relationship was showed on the left. RNA expression profile data were selected as follows: leaf of seedling stage (SS), leaf and stem of flowing period (FP), leaf and stem of mature period (MP) from YA1 (yonganxiaoye1), YA2 (yonganxiaoye2) and YA3 (yonganxiaoye3) respectively. B. Validate of candidate *BjuSPLs* in ‘YA1’ using qRT-PCR and then correlation between RNA-seq and qRT-PCR data were conducted. Each RNA-seq expression data was plotted against that from quantitative real-time PCR and fit into a linear regression. Both x- and y-axes were shown in log2 scale and each colour represented a different gene. C. Validate of candidate *BjuSPLs* in ‘YA2’ using qRT-PCR and then correlation between RNA-seq and qRT-PCR data were conducted. D. Validate of candidate *BjuSPLs* in ‘YA3’ using qRT-PCR and then correlation between RNA-seq and qRT-PCR data were conducted.

**Fig 5:**
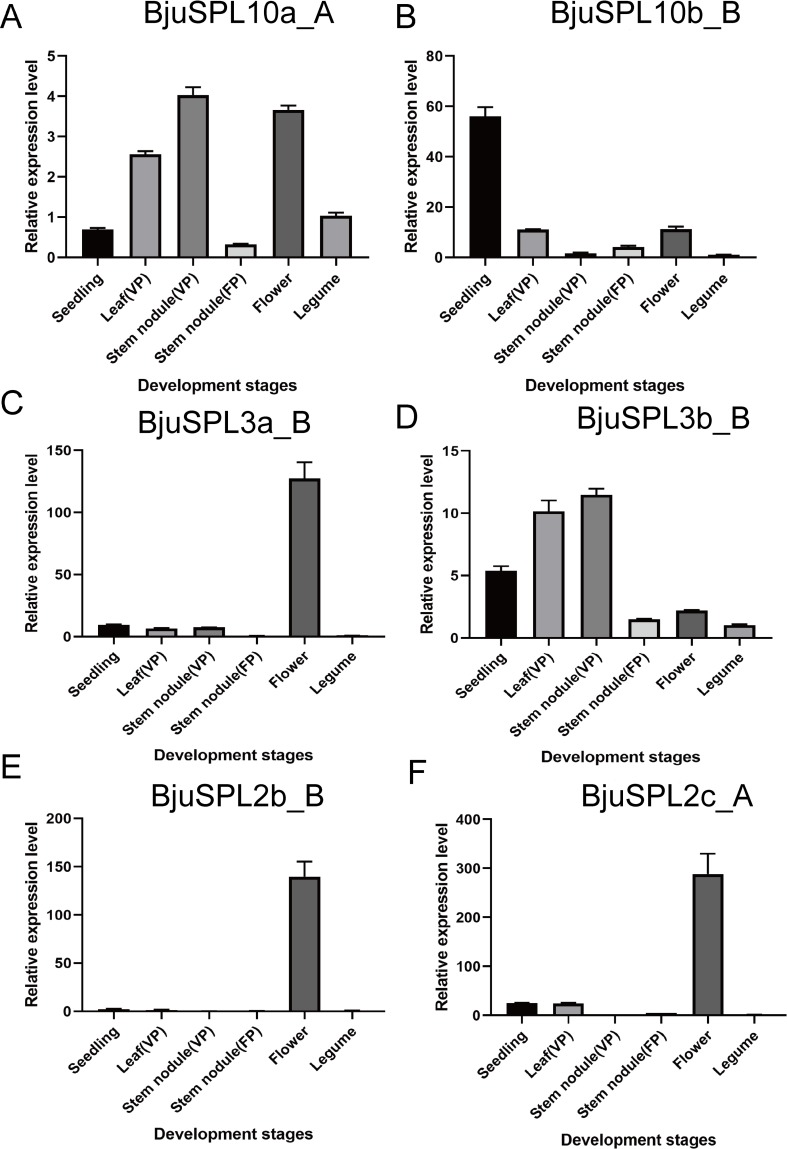
Validated of candidate *BjuSPLs* in the organ development using qRT-PCR based on their great alteration expression levels. From left to right represent seedling, leaf (Vegetative period), stem (Vegetative period), stem (Flower period), flower and legume. Error bars show the standard error calculated from three biological replicates. Values are presented as the mean ± SEM.

The expression profiles of all the identified 59 *BjuSPLs* were analyzed available from our private available RNA expression profile data. The expression of *BjuSPLs* was constructed by heat map using homemade R and showed in **Fig 4**. More interestingly, only *BjuSPL10b_A* and *BjuSPL10c_A* were found highly expressed in all tissues comprised of leaf of seedling stage (SS), leaf and stem of flowing period (FP), leaf and stem of mature period (MP) from YA1 (yonganxiaoye1), YA2 (yonganxiaoye2) and YA3 (yonganxiaoye3) respectively. Especially for *BjuSPL10c_A*, which were found highly accumulated in stem of flowing period (FP). In addition, *BjuSPL3c_A*, *BjuSPL3a_B* and *BjuSPL3b_B* were found specifically expressed in leaf of seedling stage (SS), flowing period (FP), mature period (MP) from YA2 (yonganxiaoye2) and YA3 (yonganxiaoye3), expect for YA1 (yonganxiaoye1), especially for *BjuSPL3b_B*. However, other *BjuSPL* genes were no significantly detected in all tested tissues. Expression patterns of *BjuSPL* genes with great alteration showed that *BjuSPL3a-B*, *BjuSPL2b_B* and *BjuSPL2c_A* were significantly expressed in flower; *BjuSPL 3b_B* and *BjuSPL10a_A* were significantly expressed in stem node (VP: vegetative period), but only *BjuSPL10b_B* were found cumulative in seedling (**Fig 5**).

### *MiR156*-mediated posttranscriptional regulation of *BjuSPLs*

**Fig 6:**
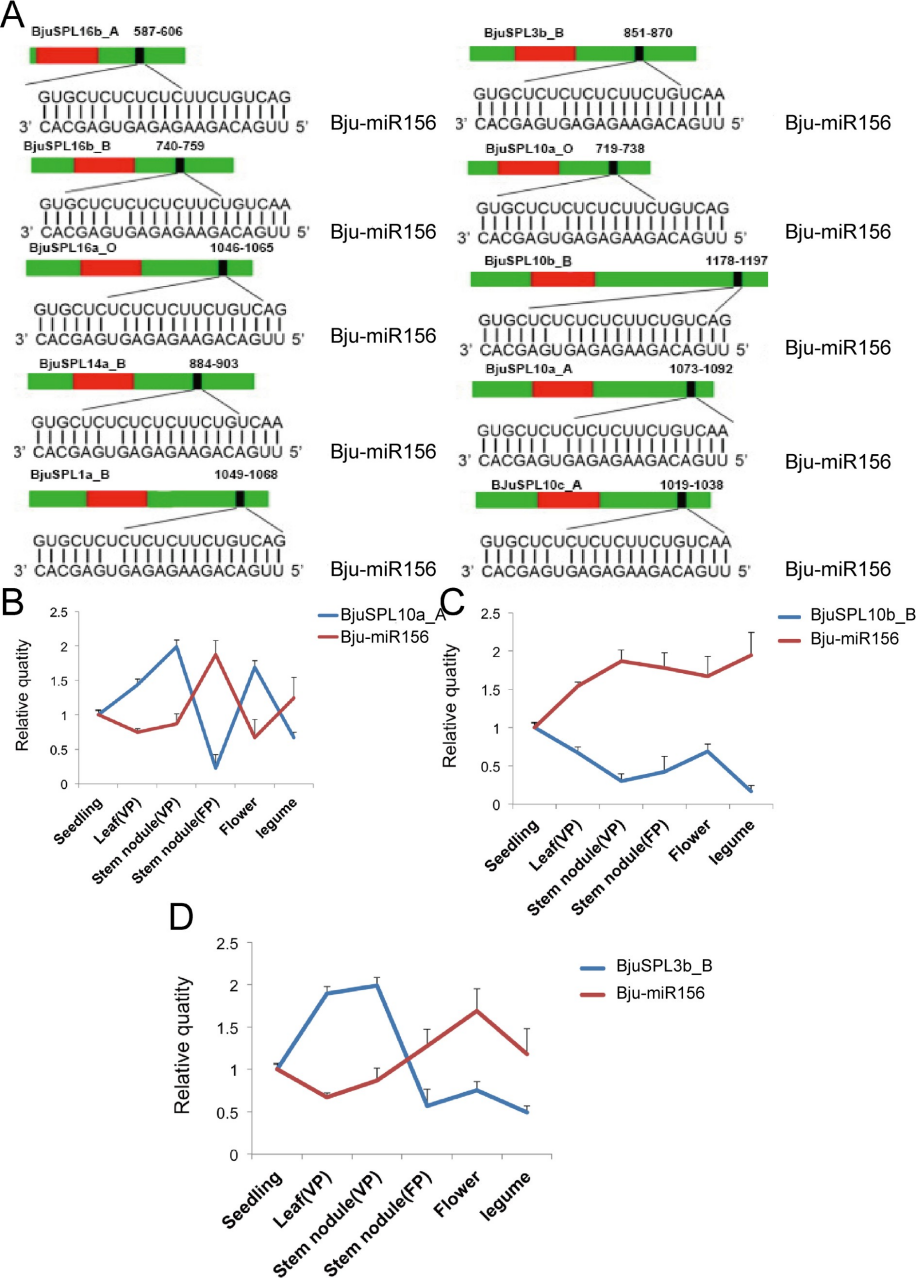
Prediction of *BjuSPLs* regulated by *miR156* and qRT-PCR analysis of miRNA156 acted in *BjuSPL10a_A*, *BjuSPL10b_B* and *BjuSPL3b_B* in *B*. *juncea*. A. Predict of *BjuSPLs* regulated by *miR156*. The miRNA target sites (red) with the nucleotide positions of *BjuSPL* transcripts are shown. The RNA sequence of each complementary site from 5′ to 3′ and the predicted miRNA sequence from 3′ to 5′ are indicated in the expanded regions. B. qRT-PCR analysis of miRNA156 acted in *BjuSPL10a_A* in *B*. *juncea*. Standard error bars are provided for three biological repeats. C. qRT-PCR analysis of miRNA156 acted in *BjuSPL10b_B* in *B*. *juncea*. D. qRT-PCR analysis of miRNA156 acted in *BjuSPL3b_B* in *B*. *juncea*.

To understand the miR156-mediated posttranscriptional regulation of the *BjuSPL* genes, we searched the coding regions and 3′ UTRs of all *BjuSPLs* for the targets of mustard *miR156a–miR156h*. The results showed that 28 *BjuSPL* genes, belonged to all groups expect for *BjuSPL9* that are complementary association with the *Bju-miR156* mature sequences **(Fig 6)**, suggesting that *Bju-miR156* may specifically regulated these *BjuSPL* genes in *B*. *juncea*. In addition, *Bju-miR156* was found acted on the location in the coding region of *BjuSPL10*, *BjuSPL11* and *BjuSPL2* belong to group I, *BjuSPL1*, *BjuSPL14* and *BjuSPL16* belong to group V and *BjuSPL3*, *BjuSPL4* and *BjuSPL5* belong to group VI, respectively. These results showed that *miR156* conserved in plants by mediated posttranscriptional regulation of the SPLs.

## Discussions

In recent years, researchers have focused on *SPL* transcription factors owing to their conservation and specially functions in plants. In addition, the number of *SPLs* have been identified varies from species to species through genome-wide analysis in plants, including 15 *SPLs* in potato [25] and tomato [26], 17–20 in rice and Arabidopsis [2, 27], as well as 27–30 in Chinese cabbage [28]. However, there is no report about *SPL* gene family in *B*. *juncea* based on genome-wide identification, owing to lack of sequenced genome information. In this research, we firstly reported systematically identification of SPL genes and investigation of their functional structure in *B*. *juncea*. Based on our results, 59 SPL genes were identified in *B*. *juncea* (**S1 Table**). Here, phylogenetic tree of *SPL* proteins in *B*. *juncea*, Arabidopsis and *B*. *napus* demonstrated that all *SPL* genes were categorized into eight groups (I–VIII) (**Fig 2**). Among 59 mustard *SPLs*, each kind of orthologs from 10 Arabidopsis *SPLs* orthologs was clustered together. In recent years, researches have demonstrated that segmental duplications were existed and play vital roles in the gene expansion of SPL transcription factor gene family in many plants [29, 30]. Here, 17 pairs of segmentally duplicated and three pairs of tandem-duplicated SPL genes were identified in the *B*. *juncea* genome. In addition, there were 12 ortholog pairs in syntenic regions between *B*. *juncea* and *B*. *napa* (S5 Table).

By conducting GSDS structure of SPLs gene in mustard, different gene structures were found in different SPL orthologs contained with the diverse number of introns, including 5 introns in *BjuSPL2*, *BjuSPL8*, *BjuSPL9*, *BjuSPL13* and *BjuSPL15*; 6 introns in *BjuSPL10* and *BjuSPL11*; 3 introns in *BjuSPL3*, *BjuSPL4* and *BjuSPL5*, as well as 12 introns in *BjuSPL1*, *BjuSPL14* and *BjuSPL16*. In addition, similar motifs were found shared in different cotton SPL orthologs through MEME analyze. 59 *BjuSPL* proteins were identified contained two Zn-finger like structure motif, and 56 BjuSPLs contained nuclear localization signal motif, except for *BjuSPL4b_A*, *BjuSPL4c_A* and *BjuSPL2B2_B*. Interestingly, most of *BjuSPL* proteins contained more motifs in SBP domain, expect for *BjuSPL15b_A* (had motif 1, 2 and 15), such as *BjuSPL2* (6–8 motifs), *BjuSPL7* (6–7 motifs), *BjuSPL14* (9–10 motifs) and *BjuSPL1* (11–13 motifs). Moreover, we found *BjuSPL1*, *BjuSPL14* and *BjuSPL16* contained 12 introns with an ankyrin (ANK) domain, suggesting that these proteins represented different functions compared with other groups. We speculated that similar function were probably shared among different SPL orthologs in mustard, based on their similar conserved motifs and exon/intron structure, expect for some specially SPL gene, such as *BjuSPL1*, *BjuSPL14* and *BjuSPL16* with an ankyrin (ANK) domain, BjuSPL4b_A, BjuSPL4c_A and BjuSPL2B2_B without nuclear localization signal, NLS.

Functional analyzed of cis-acting elements for *BjuSPLs* showed that *SPL* transcription factors may be involved in light, phytohormones and stresses responsiveness. RNA-seq profiles demonstrated that several of putative identified SPL genes show a tissue-dependent and development-dependent expression patterns (**Fig 4**). Of those, we found that *BjuSPL10b_A* and *BjuSPL10c_A* were significantly accumulated in the developmental stage and all tested tissues. *BjuSPL3c_A*, *BjuSPL3a_B* and *BjuSPL3b_B* were found specifically expressed in leaf of Seedling stage (SS), flowing period (FP), mature period (MP) from YA2 (yonganxiaoye2) and YA3 (yonganxiaoye3), expect for YA1 (yonganxiaoye1), especially for *BjuSPL3b_B*. This result suggests that only several of mustard *SPL* gene family may play vital roles in leaf and stem development, and sub-functionalization, gained new functions and lost functions possibly existed in paralog genes of *SPL* gene family. To date, less reports about expression and functional analysis of *SPL* genes were found in mustard, such as *SPL* gene family were found to be down-regulated in male-sterility lines [31] and *SPL13* was found to be cold-inducible in early stages of development. In addition, the target genes of miR156 such as *CAT*, *ABCB*, *CBS*, and *SPL* showed their involvement in stress response and temporal expression changes during leaf development in *B*. *juncea* [32–34].

We proposed that *miR156* might also involve in *SPL*-regulated gene networks in *B*. *juncea*. The previous showed that 10 *SPLs* in Arabidopsis were reported to be potential targets of *miR156/157* [5], as well as 17 *SPLs* in soybean [35], 18 *SPLs* in Populus [30] and 11 *SPLs* in rice [36]. Here, we also demonstrated that 28 *SPLs* were putative targeted by miR156. More importantly, we found that a potential target site (motif 7) was found located in those *SPLs* targeted by the *miR156*. These findings were also identified in rice and Arabidopsis, all *miR156*-targeted *SPLs* were also found *miR156* recognition element located in motif [36]. In Arabidopsis, researches have demonstrated that the most of *AtSPLs* have been acted as diverse function, such as *SPL2*, *SPL9*, *SPL10*, *SPL11*, *SPL13* and *SPL15* were specifically association with shoot development [37], *SPL3*, *SPL4* and *SPL5* primarily control flowering time [38]. *SPL3*, *SPL9* and *SPL10* specifically expressed in lateral root [39]. *SPL1* and *SPL12* acted as important regulator at the reproductive stage [40]. *SPL7* were found involved in the Cu deficiency response [41] and *SPL8* conferred to male fertility, as well as regulated gynoecium differential patterning [7, 42]. In our study, we found *BjuSPL2c_A*, *BjuSPL3a_B* and *BjuSPL2b_B* were found specifically expressed in flower in mustard and *BjuSPL10b_B* targeted by miR156 were found highly accumulated in seedling, together with *BjuSPL3b*_b and *BjuSPL10a_A* in stem node (VP). More interestingly, we demonstrated that *BjuSPL3b*_b and *BjuSPL10a_A* were negatively regulated by miR156 that specially involved in stem node (VP) development. However, the detailed expression patterns and function of those tissue-dependent and development-dependent *SPL* transcription factor, as well as their regulators miR156 remains to be further investigated in mustard. This is firstly report about identification of *SPL* gene family in mustard; it would be helpful for well functional analysis of tissue-dependent and development-dependent SPL transcription factor in the future.

## Supporting information

S1 Table Gene name and gene ID of SPLs in Arabidopsis, rice and *B*. *napa*. (XLSX)

S2 Table Expression patterns of the *BjuSPL* genes in various tissues, including leaf of seedling stage (SS), leaf and stem of flowing period (FP), leaf and stem of mature period (MP) from YA1 (yonganxiaoye1), YA2 (yonganxiaoye2) and YA3 (yonganxiaoye3) respectively. (XLSX)

S3 Table List of primers for qRT-PCR analysis of candidate *BjuSPLs*. (XLSX)

S4 Table Characterization of SPL family genes identified in *B*. *juncea*. (XLSX)

S5 Table 12 ortholog pairs in syntenic regions between *B*. *juncea* and *B*. *napa* identified using orthoVenn software. (XLSX)

S6 Table *Cis*-acting elements identified in the promoter regions of *BjuSPL* genes. (XLSX)

S1 Fig Phylogenetic tree of the *SPL* gene family in *B*. *juncea* annotated with collinear and tandem relationships. Curves connecting pairs of gene names suggest either the collinear relationship (red) or tandem relationship (blue). This annotated tree is output from ‘family tree plotter’ of MCscanX software.
(TIF)

S2 Fig Conserved motifs of *BjuSPLs* proteins identified in this study. The conserved motifs were identified using MEME (suite 4.11.4) based on the protein sequences of *BjuSPLs*, and each motif is indicated with a colored box numbered (1 to 20) at the bottom. Motif 1 and motif 2 represent two Zn-finger like structure and nuclear localization signal (NLS).
(TIF)

S3 Fig Alignment of the SBP domain in *BjuSPL* proteins. Multiple sequences alignment was performed using MAFFT version 7. Two Zn finger like structure (Cys3His-type, Cys2HisCys) and NLS are indicated. In addition, motif logo and protein sequence of the SBP domain and NLS segment were showed.
(TIF)

S4 Fig Analyze of *cis*-acting elements related to abiotic stresses and phytohormones response in *BjuSPLs* promoters. (TIF)
