# Supplementary figures and images for: Genome-wide identification and characterization, phylogenetic comparison and expression profiles of SPL transcription factor family in B. juncea (Cruciferae)
Source: PLoS One. 2019 Nov 5;14(11):e0224704. doi: 10.1371/journal.pone.0224704 (PMC6830930; doi:10.1371/journal.pone.0224704)

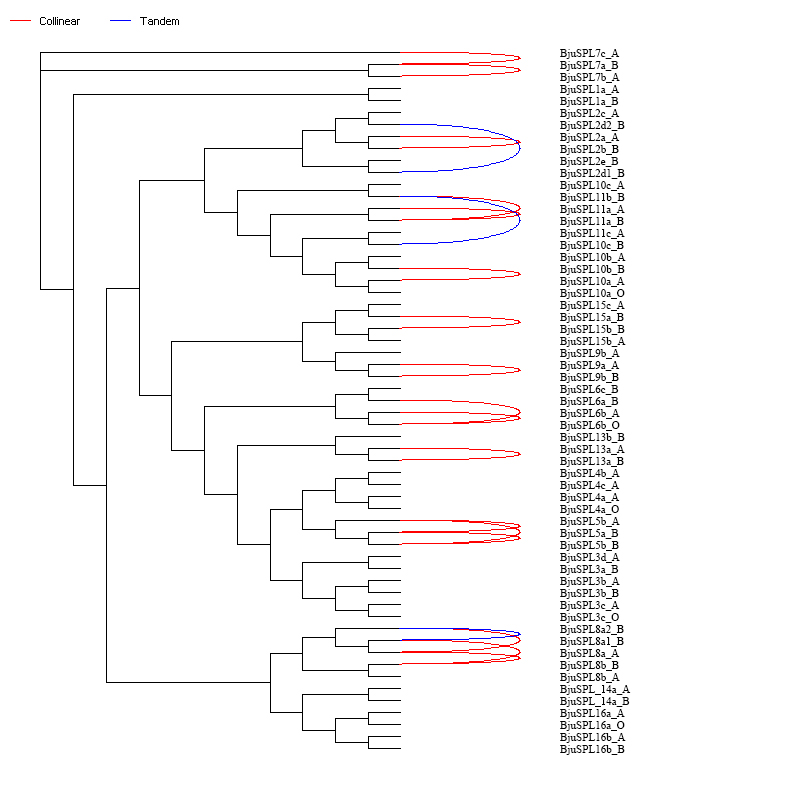

Supplement: S1 Fig — Curves connecting pairs of gene names suggest either the collinear relationship (red) or tandem relationship (blue). This annotated tree is output from ‘family tree plotter’ of MCscanX software. (TIF) [file pone.0224704.s007.tif]

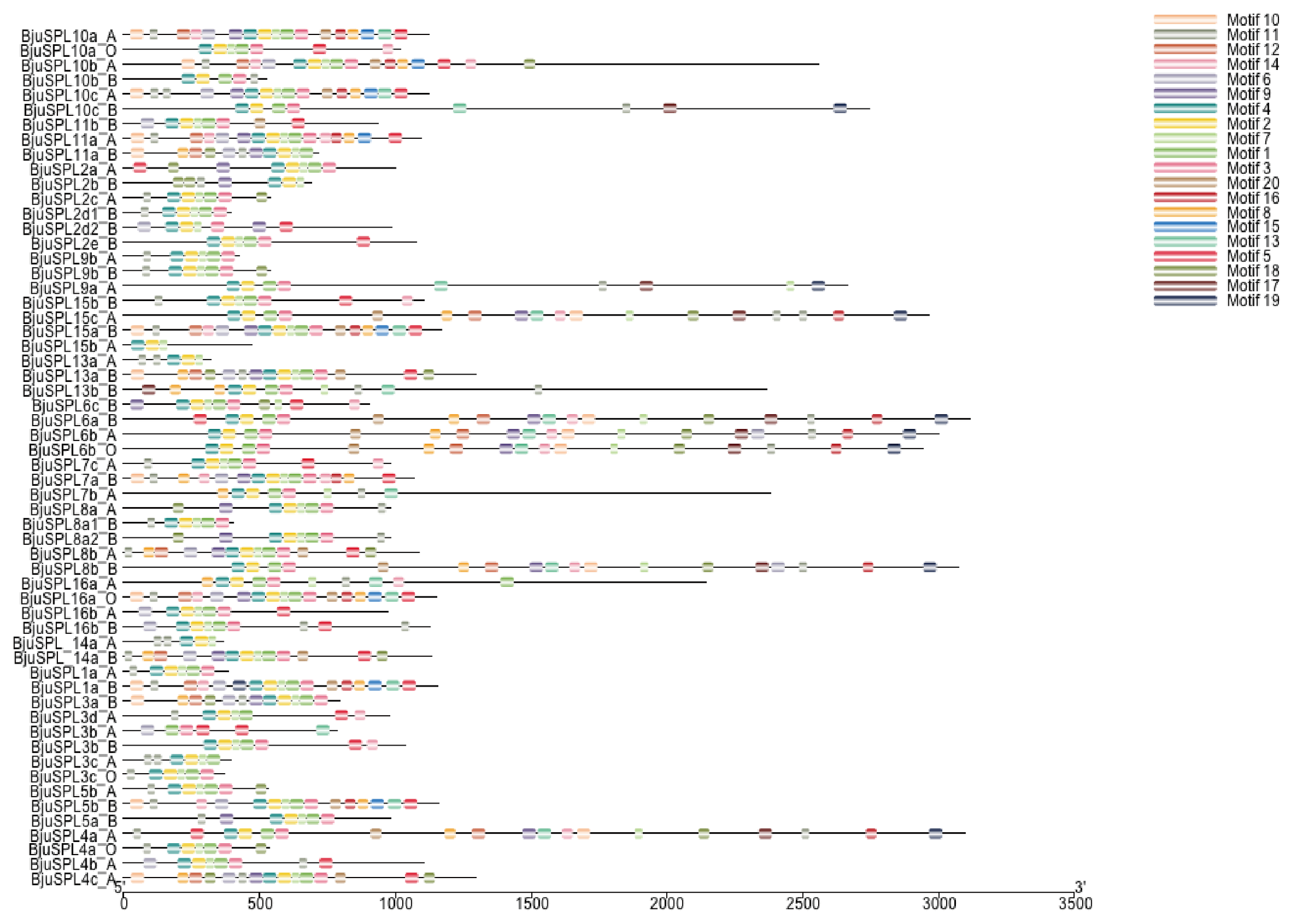

Supplement: S2 Fig — The conserved motifs were identified using MEME (suite 4.11.4) based on the protein sequences of BjuSPLs, and each motif is indicated with a colored box numbered (1 to 20) at the bottom. Motif 1 and motif 2 represent two Zn-finger like structure and nuclear localization signal (NLS). (TIF) [file pone.0224704.s008.tif]

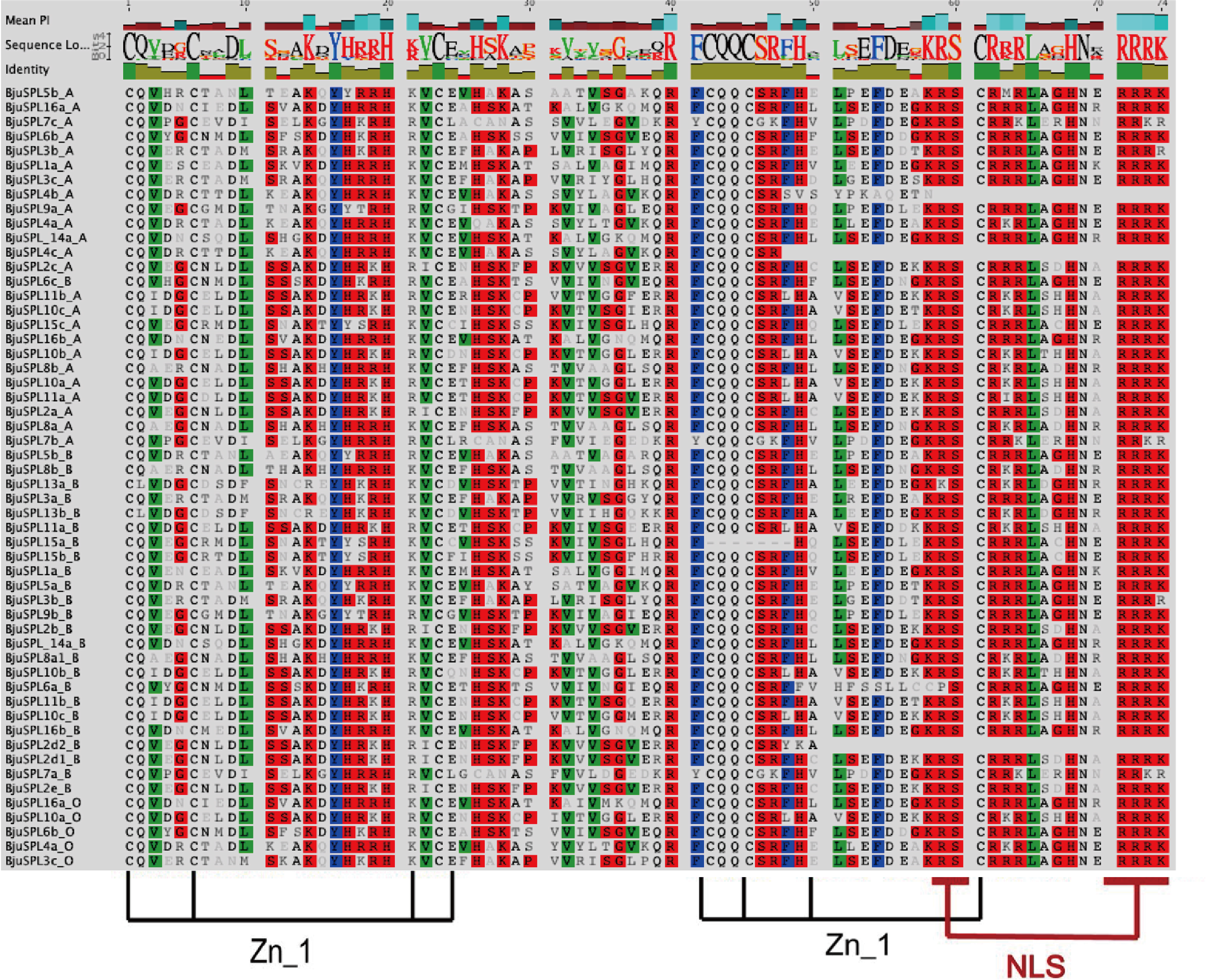

Supplement: S3 Fig — Multiple sequences alignment was performed using MAFFT version 7. Two Zn finger like structure (Cys3His-type, Cys2HisCys) and NLS are indicated. In addition, motif logo and protein sequence of the SBP domain and NLS segment were showed. (TIF) [file pone.0224704.s009.tif]

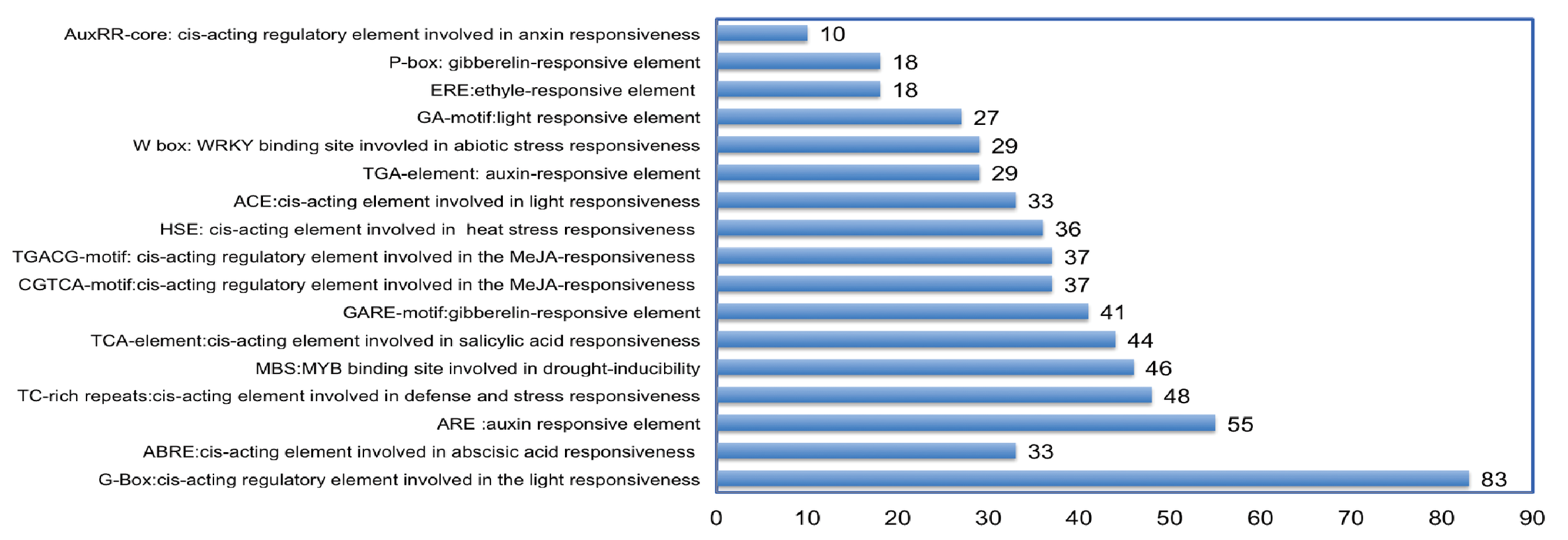

Supplement: S4 Fig — (TIF) [file pone.0224704.s010.tif]
